# Interaction of short-term depression and firing dynamics in shaping single neuron encoding

**DOI:** 10.3389/fncom.2013.00041

**Published:** 2013-04-19

**Authors:** Ashutosh Mohan, Mark D. McDonnell, Christian Stricker

**Affiliations:** ^1^The John Curtin School of Medical Research, Australian National UniversityCanberra, ACT, Australia; ^2^Computational and Theoretical Neuroscience Laboratory, Institute for Telecommunications Research, University of South AustraliaMawson Lakes, SA, Australia; ^3^Medical School, Australian National UniversityCanberra, ACT, Australia

**Keywords:** short-term depression, operating modes, emergent properties, firing properties, synaptic integration

## Abstract

We investigated how the two properties short-term synaptic depression of afferent input and postsynaptic firing dynamics combine to determine the operating mode of a neuron. While several computational roles have been ascribed to either, their interaction has not been studied. We considered two types of short-term synaptic dynamics (release-dependent and release-independent depression) and two classes of firing dynamics (regular firing and firing with spike-frequency adaptation). The input–output transformation of the four possible combinations of pre- and post-synaptic dynamics was characterized. Adapting neurons receiving input from release-dependent synapses functioned largely as coincidence detectors. The other three configurations showed properties consistent with integrators, each with distinct features. These results suggest that the operating mode of a neuron is determined by both the pre- and post-synaptic dynamics and that studying them together is necessary to understand emergent properties and their implications for neuronal coding.

## Introduction

Synapses exhibit a range of activity-dependent plasticities at various timescales (Dobrunz et al., 1997; Dittman et al., 2000; Fuhrmann et al., 2004; Regehr, 2012). Short-term synaptic plasticity is the change in efficacy of the postsynaptic potential/current upon repeated stimulation lasting for a few to hundreds of milliseconds. Excitatory synapses in neocortex exhibit short-term depression and recover at a rate of about 1 s. Depression is dominant with minimal facilitation in layers 2/3, 4, and 5 of rat barrel cortex (Cowan and Stricker, 2004; Fuhrmann et al., 2004). However, the mechanisms underlying facilitation are much less clear. Hence, we restrict our investigation to synaptic depression and its role in encoding.

Functionally, these depressing synapses show two different types of dynamics, defined here as type 1 or 2. Type 1 synapses show depression due to vesicle-depletion (VDD) that reduces the probability of neurotransmitter release upon subsequent action potentials (Markram and Tsodyks, 1996; Markram et al., 1997; Matveev and Wang, 2000; Regehr, 2012). At these synapses, the recovery rate from depression is constant. Type 1 synapses are capable of signaling a stimulus rate change but not rate (Fuhrmann et al., 2004; Jedrzejewska-Szmek and Zygierewicz, 2010). Type 2 synapses on the other hand exhibit release-independent depression, i.e., they depress even when no neurotransmitter has been released (Dobrunz et al., 1997; Thomson, 1997; Brody and Yue, 2000; Cowan and Stricker, 2004; Fuhrmann et al., 2004; Muñoz-Cuevas et al., 2004; Regehr, 2012). Additionally, the recovery rate is frequency-dependent and increases with higher stimulus frequencies (Cowan and Stricker, 2004; Fuhrmann et al., 2004). Type 2 synapses are capable of relaying both information about the stimulus rate and its rate change (Cowan and Stricker, 2004; Fuhrmann et al., 2004).

Previous work has largely focused on type 1 synapses that might endow single neurons and neuronal networks with specific capabilities. Type 1 synapses provide a gain control mechanism resulting in improved sensitivity of neurons to small changes in stimulus firing pattern (Abbott, 1997). Through simulations of networks in primary visual cortex, type 1 dynamics of thalamocortical synapses have been shown to precisely control the oscillatory response (Paik and Glaser, 2010). These properties also facilitate synchrony detection in a network (Senn et al., 1998). The functional implications of type 2 synapses have not been widely studied (but see Graham and Stricker, 2008; Scott et al., 2012). Previous studies of synaptic dynamics have primarily focused on its impact on information transfer in isolation, while neglecting the postsynaptic dynamics in detail (London et al., 2008; Fung et al., 2012).

As synaptic input is integrated at the postsynaptic side into a sequence of action potentials, the variations in firing dynamics also need consideration. The importance of studying both pre- and post-synaptic dynamics together for a holistic understanding of information processing has been recognized in the context of the dynamics of long-term plasticity and intrinsic plasticity of the postsynaptic membrane (Turrigiano et al., 1998; Xie et al., 2006; Triesch, 2007). To address this issue, we adopt the simple classification proposed by (Hodgkin, 1948)—class 1 and class 2 firing characteristics of a neuron (subsequently also called class 1 or 2 neuron). Class 1 firing is regular and there is a linear relationship between injected current and firing rate. Class 2 firing on the other hand shows spike-frequency adaption and consequently a non-linear relationship between current and firing rate. From a dynamical systems point of view, class 1 and class 2 neurons exhibit saddle node on a limit cycle and Hopf bifurcations, respectively (Izhikevich, 2000). The rationale for adopting this classification is similar to that for adopting a phenomenological description for modeling synaptic dynamics—the focus is on functional dynamics without considering the physiological mechanisms that define them.

Here, we consider all four combinations between types and classes and study how pre- and post-synaptic properties together determine whether the neuron functions as an integrator of stimuli or a coincidence detector in the presence of synaptic background noise. That the cell is quiescent with a stimulus generating sparse firing is supported by several experimental studies (Shadlen and Newsome, 1998; Brecht and Sakmann, 2002). Further, we also study how each combination is affected by variations in noise properties and extent of depression exhibited by synapses. This investigation is especially relevant in the context of highly debated question of whether neurons use precise spike timings, thereby functioning as coincidence detectors or they work more broadly using spike rates, thereby functioning as integrators (Shadlen and Newsome, 1998; deCharms and Zador, 2000). This question is also highly relevant to whether neurons are capable of acting as integrators *in vivo* where there is an increase in background conductance due to synaptic activity (Rudolph and Destexhe, 2001).

## Methods

### Stimulus

**Figure 1:**
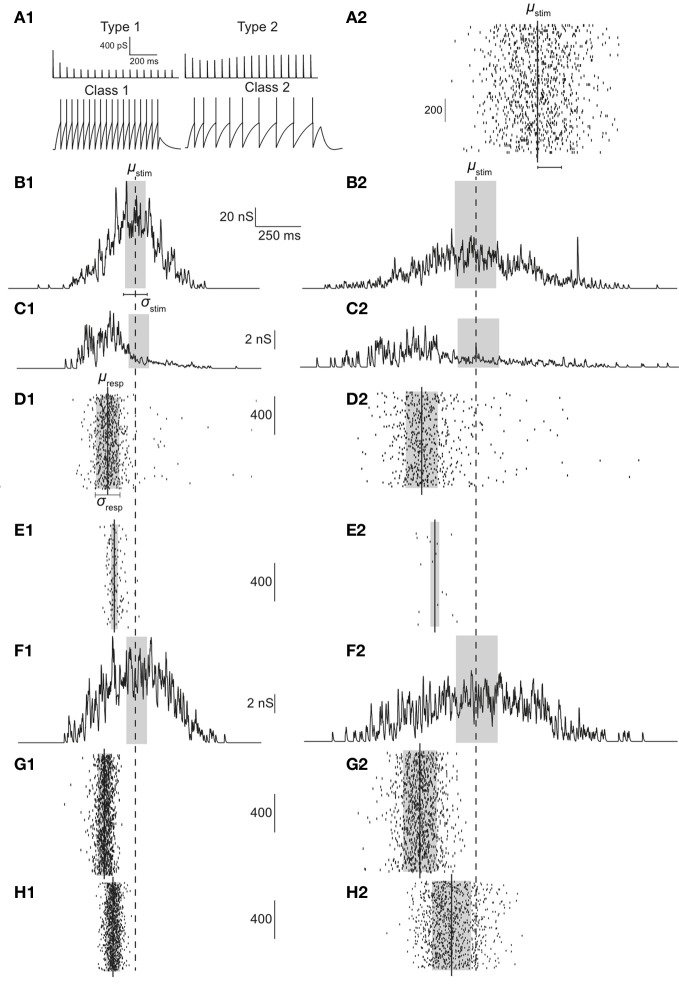
**(A1)** Response of type 1 (top left) and type 2 (top right) synapses to 30 Hz stimulation. For a current injection of 0.5 nA response of class 1 (bottom left) and class 2 (bottom right) neurons are shown. **(A2)** Illustration of stimulus. In the example, 1000 Gaussian-distributed (in time) presynaptic spikes are relayed through 75 synapses. **(B)** Synaptic conductance if synapses were static; σ_stim_ = 60 **(B1)** or 120 ms **(B2)**. **(C,F)** Synaptic conductances with type 1 **(C)** synapses and type 2 **(F)** synapses. **(D,E)** With type 1 synapses, raster of spiking response over 1000 iterations for class 1 **(D)** and class 2 **(E)** neurons. **(G,H)** With type 2 synapses, raster of spiking response over 1000 iterations for class 1 **(G)** and class 2 **(H)** neurons. Dashed line signifies stimulus mean (μ_stim_) while solid lines indicate response mean (μ_resp_). Gray boxes indicate ±0.5·σ_stim_ in (**B,C**, and **F**) and ±0.5·σ_resp_ in (**D,E,G**, and **H**).

Each stimulus consisted of *N*_tot_ number of presynaptic spikes delivered through *N*_syn_ number of synapses (either type 1 or 2) that relay excitatory postsynaptic potentials to the postsynaptic neuron with either class 1 or 2 firing characteristics. As shown in Figure 1, this stimulus was constructed as follows. *N*_tot_ Gaussian random numbers were generated with the specified parameters. Each of the generated numbers was assigned to a randomly picked synapse. The sum of all synaptic stimulations, thus, had a Gaussian distribution (in time). Simulations were performed by repeated iterations using a Gaussian stimulus, which was computed by distributing *N*_tot_ stimuli across *N*_syn_ number of synapses (see Figure 1A2). The timing of each presynaptic spike that comprises the stimulus was based on a Gaussian distribution with the following two parameters, μ_stim_ and σ_stim_ where the former is the mean of the stimulus distribution and the latter its standard deviation, subsequently also called dispersion. Specifically, since presynaptic spike times are generated based on a Gaussian distribution, this parameter signifies the time of stimulus peak. Small values of σ_stim_ imply tightly synchronized presynaptic spike arrivals while large values imply a less synchronized stimulus.

In order to facilitate comparison and interpretation of various values, σ_stim_ and, in general, all values capturing a time quantity were normalized by the membrane time constant τ_*m*_. As an example, if σ_stim_ = 0.1, dispersion of the stimulus is 10% of the time constant. Since in a Gaussian distribution, 99.73% of all events occur within three times the standard deviation on either side of the mean, this implies that almost all presynaptic spikes arrive within 60% of τ_*m*_.

#### Synapse model

The phenomenological model used is an extension of that proposed by Fuhrmann et al. (2004). Type 1 synapses show release-dependent depression with a constant rate of recovery. Type 2 synapses show release-independent depression and a frequency-dependent recovery rate. The model exhibits either type 1 or 2 dynamics depending on the parameter values.

The synaptic conductance (*g*_*s*_) due to a single synapse is computed as:

$$g_{s}(t)=U_{\text{SE}}(t)\cdot P_{v}(t)\cdot A_{\text{SE}}$$

*U*_SE_ and *P*_*V*_ represent the maximal response when all synapses release their vesicles and probability of vesicle availability, respectively. Their product corresponds to the fraction of available vesicles that are released. *A*_SE_ is the maximal conductance. The variables in turn are governed by the following set of equations. The first is,

$$\frac{dP_{V}}{dt}=\frac{1-P_{V}}{\tau_{VDD}}-U_{SE}\cdot P_{V}\cdot \sum_{N_{tot}}\delta \left(t-t_{AP}\right),$$

where τ_VDD_ is the time constant of the synaptic vesicle refilling process, δ is the Dirac delta function and *t*_AP_ is the time of arrival of an action potential. The formulation of release-independent depression is encapsulated with the variable *U*_SE_ being decremented from an initial availability of *U*_0_ with a strength of *S*_RID_ followed by an exponential recovery with a characteristic time constant τ_RID_, i.e.,

$$\frac{dU_{\text{SE}}}{dt}=\frac{U_{0}-U_{\text{SE}}}{\tau_{\text{RID}}}-S_{\text{RID}}\cdot U_{\text{SE}}\cdot \sum_{N_{\text{tot}}}\delta (t-t_{\text{AP}}).$$

In analogy, the frequency-dependent recovery of type 2 synapses is captured by decrementing the recovery time constant with a strength of *S*_FDR_ upon the arrival of an action potential, i.e.,

$$\frac{d\tau_{\text{RID}}}{dt}=\frac{\tau_{0}-\tau_{\text{RID}}}{\tau_{\text{FDR}}}-S_{\text{FDR}}\cdot \tau_{\text{RID}}\cdot \sum_{N_{\text{tot}}}\delta (t-t_{\text{AP}}).$$

In other words, the recovery rate becomes faster following which τ_RID_ approaches its original value with an exponential time course governed by τ_FDR_.

For excitatory synapses, typical model parameter values of type 1 and 2 synapses were chosen based on parameter estimates using experimental data of Fuhrmann et al. (2004).

The model has six parameters with values as specified in Table 1.

**Table 1:** **Synapse parameters**.

**Parameter/Type**	**Type 1**	**Type 2**
*U*_0_	0.6	0.25
τ_VDD_ [s]	0.5	0.005
τ_FDR_ [s]	0.9	0.9
τ_0_ [s]	0.6	0.6
*S*_RID_	0	0.25
*S*_FDR_	0	0.30
*A*_SE_ [nS]	1	1

#### Noise model

A noisy current *I*_*N*_, was injected into neurons and modeled as an Ornstein–Uhlenbeck process (OUP) and approximated in discrete time simulations using the method proposed by Gillespie (1996), i.e.,

$$I_{N}\left(n\right)=\left(1-\frac{\Delta t}{\tau_{N}}\right)\cdot I_{N}\left(n-1\right)+\left(\sigma_{N}\sqrt{\frac{2\Delta t}{\tau_{N}}}\right)G\left(0,1\right)\text{​},$$

where *G*(0,1) is a zero mean, unit variance Gaussian distributed number. The sample time Δτ was set to 0.2 ms. This noise is characterized by the standard deviation (σ_*N*_) and the correlation time (τ_*N*_) which indicates the time window within which correlations in noise can be observed. As no two samples of white noise are correlated, an increase in the correlation time window results in greater “coloring” of white noise. τ_*N*_ was varied to study how it interacted with short-term synaptic dynamics in shaping the neuronal response properties. The standard deviation of the process σ_*N*_ was set to a constant value of 50 pA and τ_*N*_ was varied in the simulations. Action potentials generated were almost always due to the stimulus and very rarely sole due to injected noise (<1%).

### Response

Each Gaussian stimulus comprising of several presynaptic spikes was relayed to the postsynaptic neuron through dynamic synapses. To explore the operating mode of the neuron, *N*_syn_ was varied between 75 and 125 in steps of 5 and the background noise correlation τ_*n*_ was varied between 50 and 100 in steps of 10. *N*_tot_ was set to 1000, unless mentioned otherwise. For each parameter set, individual Gaussian stimuli were repeated 5000 times and if the neuron spiked, the time of the first action potential was recorded. Resulting peri-stimulus time histograms (PSTHs) were characterized by a Gaussian distribution of width σ_resp_ and with respect to the stimulus distribution, shifted by a precession, *t*_pre_ (see Figure 1). Timing of only the first action potential was considered. While acknowledging the potential of spike trains to encode information, the focus of this study is on the encoding of stimulus information in the timing, reliability, and dispersion of the first action potential. Information encoded in repeated spiking is not considered.

#### Neuron model

We used an adaptive integrate-and-fire model formulated by Brette and Gerstner (2005); i.e.,

$$C\frac{dV}{dt}=f(V)-I_{W}(t)-I_{N}(t)-g_{\text{S}}(t)\cdot (V-E_{e}),$$

where *V* is membrane voltage, *C* is the membrane capacitance, *f*(*V*) the function capturing the passive properties and the action potential generation dynamics, *I*_*w*_ the adaptation current, *I*_*N*_ the injected noise, *g*_S_ the synaptic conductance, and *E*_e_ the reversal potential for excitatory synapses. *f*(*V*) is defined as:

$$f(V)=-g_{L}\cdot (V-E_{L})+g_{L}\cdot \Delta_{T}\cdot \exp\left(\frac{V-V_{T}}{\Delta_{T}}\right)\text{​},$$

where *g*_*L*_ is the leak conductance, *E*_*L*_ the leak reversal, Δ_*T*_ the slope factor, and *V*_*T*_ the spike threshold.

The adaptation current, *I*_*W*_, is generated as follows:

$$\tau_{W}\frac{dI_{W}}{dt}=a\cdot \left(V-E_{L}\right)-I_{W},$$

where τ_*w*_ is the time constant determining the rate of spike frequency adaptation. When an action potential is generated and the membrane potential (*V*) goes over the threshold (*V*_*T*_):

$$\begin{array}{l} \;V\to E_{L}\\ I_{w}\to I_{w}+b \end{array}$$

where *b* represents spike-triggered adaptation.

For class 1 neurons, the parameters were exactly those specified in Brette and Gerstner (2005), except that for class 1 and 2, *a* was set to 1 and 8, respectively. The variable that mainly determines the class is the subthreshold adaptation variable *a* with the spike-triggered adaptation variable *b* playing a more minor role in our simulations (Touboul and Brette, 2008). See Table 2 for parameter values of the neuron models.

**Table 2:** **Neuron parameters**.

*C* [pF]	1000
*g*_*L*_ [nS]	8
*E*_*L*_ [mV]	−70.6
*V*_*T*_ [mV]	−50.4
Δ_*T*_ [mV]	2
τ_*W*_ [ms]	144
*a* [nS]	1 (class 1) or 8 (class 2)
*b* [nA]	0.0805

#### Response characteristics

We define the following variables that capture the characteristics of the spiking response, namely *N*_iter_ as the total number of iterations (set to 5000 in our simulations), *N*_resp_ as the number of spikes evoked over all iterations, *R* as the reliability of spike generation, defined as the ratio of number of spikes evoked across all iterations and the total number of iterations; i.e., *R* = *N*_resp_/*N*_iter,_ *t*_pre_ as the precession of the mean of response Gaussian distribution with respect to the stimulus distribution, normalized by the membrane time constant τ_*m*_, σ_resp_ as the width of the response Gaussian distribution, again normalized by τ_*m*_ and ζ as the sharpening of responses defined as the ratio between the stimulus and response dispersions (σ_stim_/σ_resp_).

#### Definition of operating modes

We considered the two operating modes coincidence detector and integrator. As an operational definition, we defined each mode in terms of one or more response parameters. Coincidence detectors were defined to be reliable (*R* > 0.75) only for tightly synchronized stimuli (defined as, σ_stim_/τ_*m*_ < 0.4) and otherwise unreliable (*R* = 0.75). Thus, a coincidence detector is selectively sensitive to synchronized inputs while failing to reliably relay dispersed inputs. Integrators were defined as being reliable over a range of stimulus synchronies (0.2 < σ_stim_/τ_*m*_ < 1.2) but requiring to exhibit a regular relationship between stimulus and response dispersion. Thus, an integrator relays stimulus information reliably with the response dispersion having a regular relationship with stimulus dispersion.

#### Simulation

All simulations were done in Igor Pro 6.2 (WaveMetrics Inc., Lake Oswego, OR, USA) on a Windows 7 workstation. For the synapse model, the analytic solution was used instead of solving the differential equations (Scott et al., 2012). For the neuron model, the differential equations were solved numerically using a fourth order Runge–Kutta algorithm (Press et al., 2007). Five thousand trials took approximately 1 h with a time step Δτ = 0.2 ms. All analysis was done using custom routines written in Igor Pro.

## Results

Type 1 synapses show release-dependent depression and constant recovery rate while type 2 synapses show release-independent depression with a faster recovery rate for higher presynaptic spike rates. Figure 1A1 (top) shows these two examples stimulated at 25 Hz. Type 1 synapses depress and rapidly reach the steady state, the amplitude of which is inversely proportional to the stimulus frequency (Cowan and Stricker, 2004; Fuhrmann et al., 2004). Type 2 synapses on the other hand, depress but also recover rapidly and hence exhibit a larger steady state response the amplitude of which is more or less constant. Thus, it might be expected that type 1 synapses are effective to relay low frequency stimuli or high frequency stimuli that are highly synchronous. Type 2 synapses might be expected to be able to relay low and high frequency stimuli irrespective of the degree of synchronization.

On the postsynaptic side, class 1 neurons fire regularly and class 2 neurons show spike-frequency adaptation (see Figure 1A1; bottom). The ability to generate the first action potential is higher for class 2 neurons as the dynamics enables firing at arbitrarily low frequencies. Thus, class 1 neurons might be expected to be able to relay incoming stimuli irrespective of the degree of synchronization. Class 2 neurons cannot relay highly synchronized (i.e., not dispersed) inputs because the latter cannot depolarize the membrane sufficiently enough to counteract the hyperpolarizing current present in class 2 neurons.

**Figure 2:**
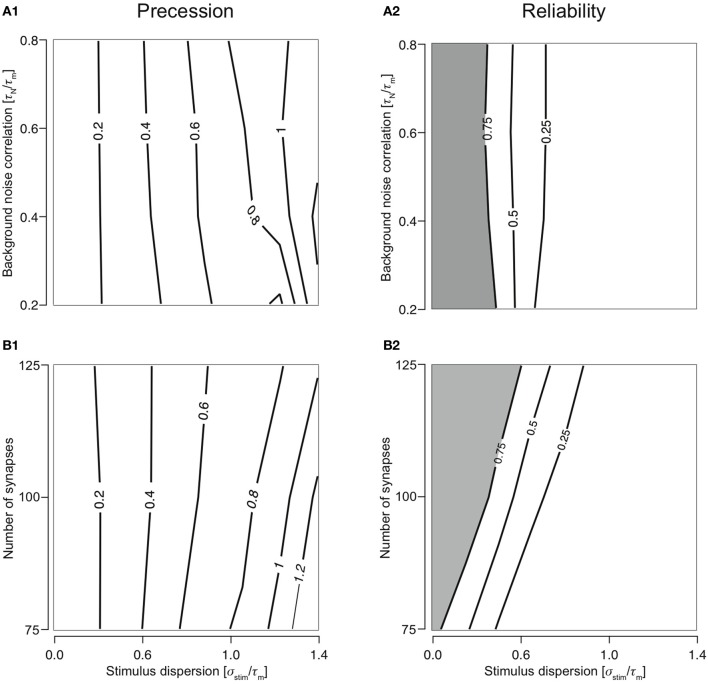
**Cellular response with varying number of synapses and background noise correlation. (A)** Contour plots showing precession **(A1)** and reliability **(A2)** of response Gaussian distribution with respect to the stimulus distribution and changing background noise correlation (*N*_syn_ set to 100). Contour lines join points of equal value thus indicating regions in the two-dimensional parameter space (stimulus synchrony vs. noise correlation) in which an response characteristic of the system is similar even while parameter values change. In addition, contour lines are useful in visualizing regions which are lesser or greater than a specified value. **(B)** Contour plots showing precession **(B1)** and reliability **(B2)** of response Gaussian distribution with respect to the stimulus distribution and synapse number (τ_*N*_ is set to 50 ms).

Both synaptic and postsynaptic dynamics have implications in how presynaptic spike information is processed. This is illustrated in Figures 1C–H. As shown in Figure 1C, when a Gaussian stimulus is transmitted through type 1 synapses, the peak of the stimulus is shifted to the left (precession) in addition to a general decrease in amplitude due to depression. No such precession is observed with type 2 synapses (Figure 1F), which also depress less. As a result, even if the stimulus arriving from presynaptic neurons is the same, the response of class 1 differs depending on whether the stimulus is transmitted through type 1 (Figure 1D) or 2 synapses (Figure 1G). Similarly, the response of class 2 neurons differs based on whether the stimulus is transmitted via type 1 (Figure 1E) or 2 synapses (Figure 1H).

We systematically investigated the operating mode of a neuron for all possible combinations between synaptic types (T) and firing class (C); i.e., T1C1, T1C2, T2C1, and T2C2. In addition, the impact of the number of synapses comprising the stimulus and the injected background noise correlation was also studied. The number of synapses was chosen as a parameter because the extent of the number of synapses influences the amount of synaptic depression. The background noise correlation was included in order to study the interaction with the time constants of synaptic depression and recovery.

### T1C2 allows for coincidence detection

As predicted, the response of a neuron with class 2 firing receiving inputs through type 1 synapses is largely reliable for highly synchronous stimuli (smaller σ_stim_). A reliable response is, by definition, when *R* > 0.75 (shaded regions in Figures 2A2 and 2B2). As the stimulus becomes more dispersed (increasing σ_stim_), reliability decreases rapidly. This property is robust to variations in noise correlation and number of synapses. Dispersion of stimulus largely determines response precession (Figures 2A1 and 2B1). This property is also robust to variations in noise correlation and number of synapses for stimulus dispersion, σ_stim_ <0.8.

Varying the synapse number while keeping τ_*n*_ to 50 ms reveals the extent to which presynaptic depression dynamics shape the response properties of the neuron. For example, if *N*_tot_ = *N*_syn_, each synapse will, on average contribute only one event to the total stimulus. Since the first response of all synapses is identical and depression is apparent only from the second stimulus onwards, no effects of depression can be observed in this case. As the value of *N*_syn_ is decreased, each synapse receives a greater number of presynaptic spikes to the total stimulus and hence, the responses are subject to more depression. With changing synapse number, the ability for coincidence detection of the T1C2 configuration remains unaltered. Precession is largely determined by the stimulus dispersion (Figure 2B1). However, reliability is dependent on the number of synapses (Figure 2B2). A decrease in the number of synapses (increase in number of presynaptic spikes delivered to each synapse) results in greater overall depression and hence reduces reliability.

### Remainder of the configurations are largely integrators

**Figure 3:**
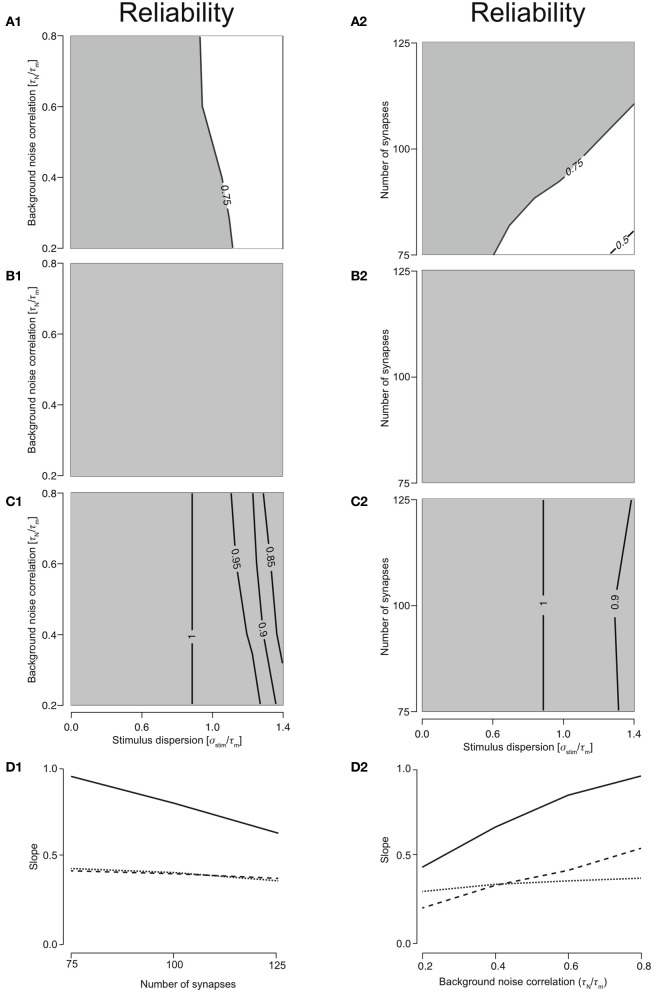
**Reliability of the cellular response with varying number of synapses and background noise correlation. (A)** Contour plots reliability for varying noise correlation **(A1)** and number of synapses **(A2)** for class 1-type 1 configuration. **(B)** Contour plots showing reliability for varying noise correlation **(B1)** and number of synapses **(B2)** for class 2-type 1 configuration. **(C)** Contour plots showing reliability for varying noise correlation **(C1)** and number of synapses **(C2)** for class 2-type 2 configurations. In all of the above contour plots, when noise correlation is varied, *N*_syn_ is set to 1000 and when number of synapses is varied, τ_*N*_ is set to 50 ms. **(D)** Analysis of the slope of relationship between stimulus synchrony and response jitter. Graphs are plotted with the number of synapses **(D1)** or the noise correlation **(D2)** systematically changing along the abscissa with the corresponding slope plotted along the ordinate. **(D1)** For *N*_syn_ = 100 and τ_*N*_ = 50, plot showing the relationship between number of synapses and ratio between response and stimulus dispersion. A straight line was fit and the slope computed. This was repeated for all parameter values to obtain relationship between number of synapses and the slope **(D2)** for T1C1 (solid), T2C1 (dashed), and T2C2 (dotted).

Responses were reliable (*R* > 0.75) through out the range of simulated stimulus dispersions (0.1–1.4) for T1C1, T2C1, and T2C2 configurations. For T1C1, the reliability was primarily determined by the stimulus dispersion when the noise correlation was varied, keeping *N*_syn_ = 1.0 (Figure 3A1). Moreover, reliability did not decrease dramatically as demonstrated by T1C2 configuration, i.e., the coincidence detector. For varying number of synapses (with τ_*N*_ = 50 ms), the reliability was determined by the stimulus dispersion and the number of synapses. As might be expected with an increasing number of synapses, reliability drops slightly (Figure 3A2) due to increased depression of type 1 synapses. Simultaneously increasing stimulus dispersion also improves reliability of response.

For integrators, an increase in stimulus dispersion must result in an increase in response jitter. We investigated this by computing the slope of this relation for various parameters. The relation between stimulus dispersion and response jitter was always more or less linear with varying slopes. For various values of noise correlation and synapse number, we computed the slope and plotted them against noise correlation (Figure 3D1) and number of synapses (Figure 3D2). T2C1 and T2C2 exhibited more or less similar slopes. Given that type 1 synapses depress rapidly, a surprising result was that the T1C1 configuration exhibited the steepest slope. This suggests that both pre- and postsynaptic dynamics together determine the operating mode of the neuron.

### T1C1: preserves synchrony most effectively

**Figure 4:**
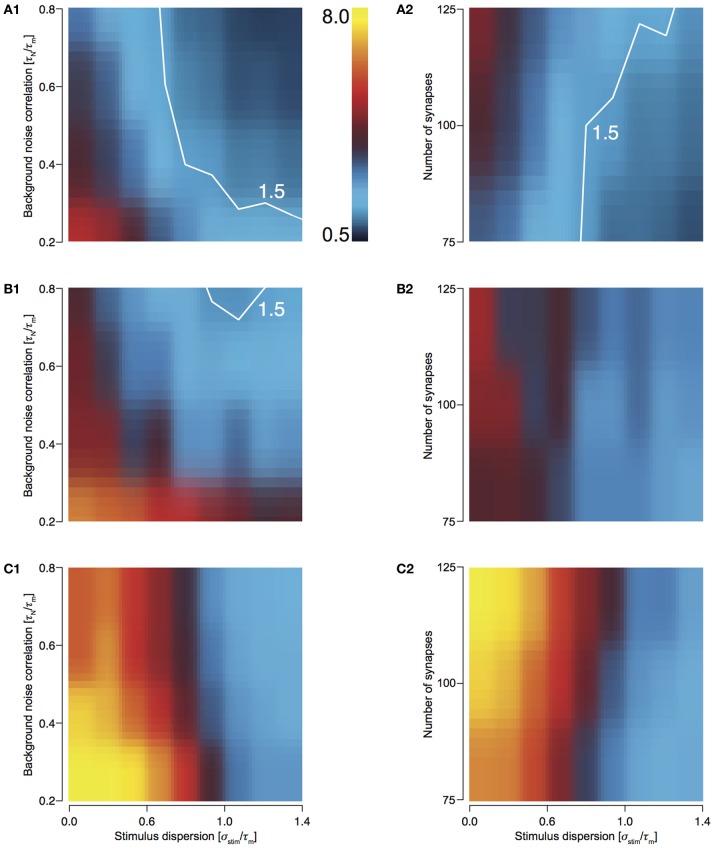
**Sharpening of the cellular response with varying number of synapses and background noise correlation. (A–C)** Heat plots showing response sharpening for varying noise correlation **(A1)** and number of synapses **(A2)** for T1C1, T2C1, (**B1** and **B2**), and T2C2 (**C1** and **C2**) configurations. In all of the above heat plots, when noise correlation is varied, *N*_syn_ is set to 100 and when number of synapses is varied, τ_*N*_ is set to 50 ms. Scaling of the heat plot is linear from values of 0.5 to 8. The white line in (**A1,A2**, and **B1**) is an isocline with a value of 1.5. The area circumscribed by this isocline encompasses sharpening values less than or equal to 1.5. The corresponding area in the other graphs is negligible.

To study how the four configurations preserve stimulus synchrony in their response jitter, we investigated the behavior of response sharpening, ξ = σ _stim_/σ_resp_. Strictly speaking if ξ < 1, the response of the neuron does not preserve stimulus synchrony. Instead, the response jitter is more desynchronized than the stimulus. If ξ = 1, stimulus synchrony is preserved. If ξ > 1, response synchronization is greater than that of the stimulus; i.e., synchrony is enhanced. We define the region 0.5 < ξ < 1.5 as preserving the stimulus synchrony in the response jitter. For T1C1 configuration, this region is larger (Figures 4A1 and 4A2) than for T2C1 (Figures 4B1 and 4B2) and T2C2 configurations (Figures 4C1 and 4C2). For T2C1, the area is least compared to the other two configurations. T2C2 shows the highest sharpening, which is robust to variations in noise correlation (Figure 4C1) and number of synapses (Figure 4C2). This is consistent with previous work (Pinto et al., 1996; Marella and Ermentrout, 2008), which suggests that class 2 neurons show a greater tendency toward stochastic synchronization than class 1 neurons.

T1C1 neurons show the greatest preservation of stimulus synchrony, especially as dispersion of stimulus increases. An increase time constant of noise correlation results in an increase in the preservation of synchrony (ξ tends toward 1 or lower).

**Figure 5:**
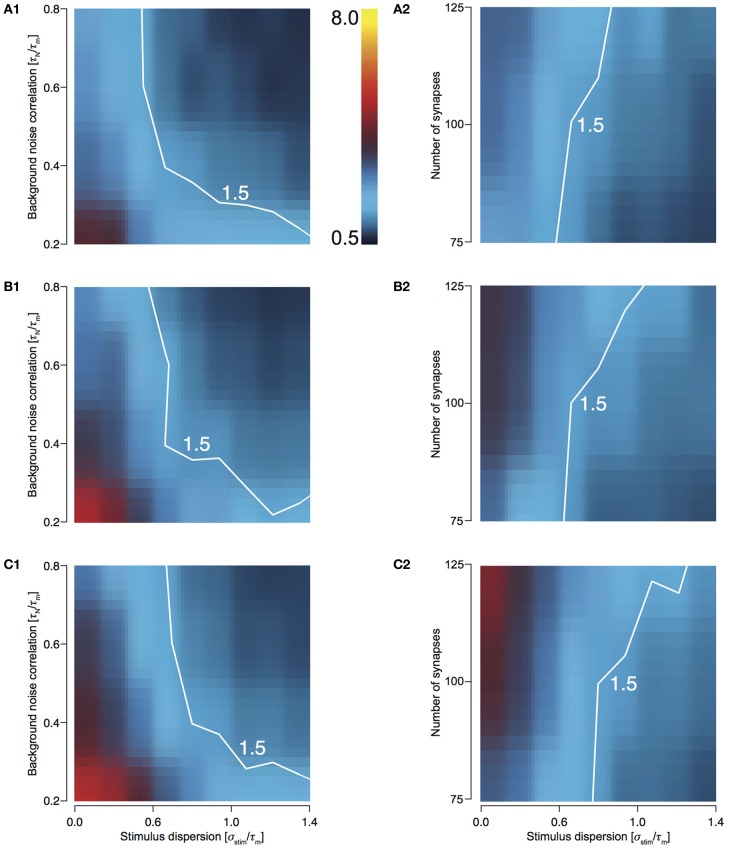
**Sharpening of the cellular response of T1C1 with varying number of synapses and background noise correlation. (A–C)** Heat plots showing sharpening for varying noise correlation **(A1)** and number of synapses **(A2)** when total number of presynaptic spikes (*N*_tot_; see Methods) was set to 500, 750 (**B1** and **B2**), and 1000 (**C1** and **C2**). In all of the above heat plots, when noise correlation is varied, *N*_syn_ is set to 100 and when number of synapses is varied, τ_*N*_ is set to 50 ms. Scaling of the heat plot is linear from values of 0.5 to 8. The white line in all the above graphs is an isocline with a value of 1.5. The area circumscribed by this isocline encompasses sharpening values less than or equal to 1.5.

In order to explore the preservation of stimulus dispersion by T1C1, we studied the behavior of response sharpening (ξ) for three different total numbers of presynaptic spikes, *N*_tot_ = 500, 750, and 1000. As the total number of spikes increases, the area under the contour indicating synchrony preservation progressively decreases. For 500 stimuli, this area is largest (Figures 5A1 and 5A2) with the area decreasing for 750 (Figures 5B1 and 5B2) and even more for 1000 (Figures 5C1 and 5C2). For highly synchronized stimuli (σ_stim_/τ_*m*_ < 0.5), synchrony preservation was primarily determined by noise correlation and only to a much lesser extent by the number of synapses comprising the total stimulus. Type 1 synapses depress rapidly, especially when relaying highly synchronous stimuli at a high frequency. Thus, the response to a change in stimulus to the neuron after depression is minimal and hence it has little effect on synchrony preservation. But for a less synchronous stimulus, preservation of synchrony is dependent on the number of synapses. Type 1 synapses are in a less depressed state and hence small changes in synchrony are relayed to the postsynaptic neuron. Note that even though the area indicating synchrony preservation varies for different number of stimuli, the maximum sharpening for highly synchronous stimuli remains roughly the same (3.2–3.6). This suggests that for a small number of stimuli, synchrony preservation is more robust to variations in noise correlations and number of synapses.

### T2C1: most reliable integrator

For T2C1, responses were always reliable (*R* = 1) when either noise correlation or number of synapses was varied (Figures 3B1 and 3B2). This is explained by the fact that type 2 depress less than type 1 synapses. Moreover, they undergo frequency-dependent recovery and hence are much more capable of reliably relaying presynaptic spikes to the neuron. But this property is not entirely dependent on synapse type alone. For T2C2, responses were reliable (*R* > 0.75) when noise correlation or number of synapses was varied (Figures 3C1 and 3C2). But reliability is not as perfect as with class 1 neurons. This is because class 2 neurons have a hyperpolarizing current, which reduces the firing an action potential; i.e., reliability. Thus, while synapses with smaller depression can influence a configuration to function as an integrator, synapse type alone does not govern operating mode. For example, class 2 neurons receiving type 1 synapses function as coincidence detectors (see above), but when class 1 neurons receive type 1 synapses, the operating mode is that of an integrator. Thus, operating mode of a configuration is set synergistically by both synaptic and neuronal dynamics.

### T2C2: maximum response sharpening

**Figure 6:**
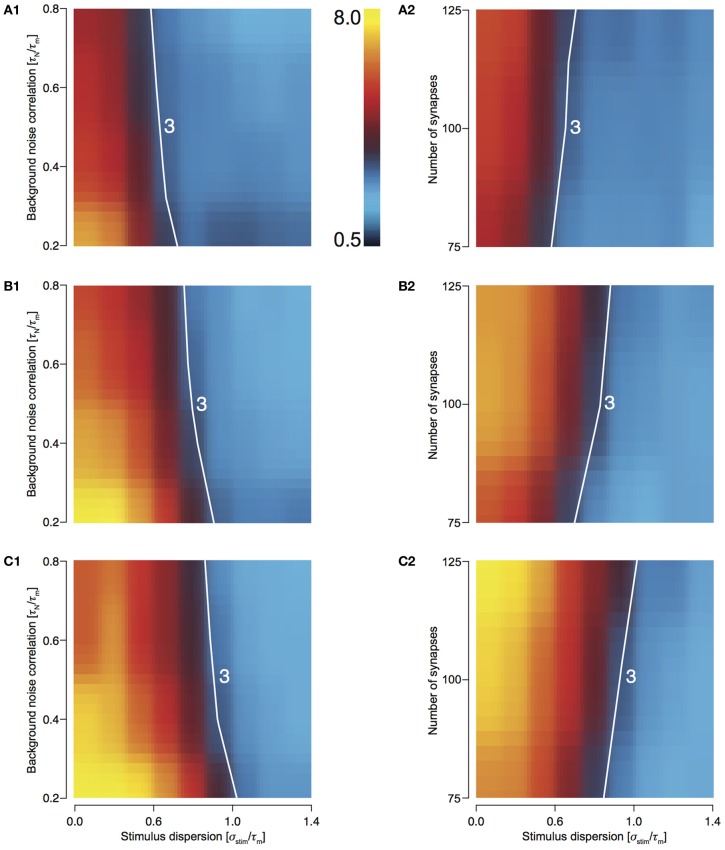
**Sharpening of the cellular response of T2C2 with varying number of synapses and background noise correlation. (A–C)** Heat plots showing sharpening for varying noise correlation **(A1)** and number of synapses **(A2)** when total number of presynaptic spikes (*N*_tot_; see Methods) was set to 500, 750, (**B1** and **B2**) and 1000 (**C1** and **C2**). In all of the above heat plots, when noise correlation is varied, *N*_syn_ is set to 100 and when number of synapses is varied, τ_*N*_ is set to 50 ms. Scaling of the heat plot is linear from values of 0.5 to 8. The white line in all the above graphs is an isocline with a value of 3.0. The area circumscribed by this isocline encompasses sharpening values less than or equal to 3.0.

For T2C2, we studied the behavior of response sharpening (ξ) for three different total numbers of presynaptic spikes, *N*_tot_—500, 750, and 1000 (see Methods). For 500 stimuli, this area is smallest (Figures 6A1 and 6A2) and increasing for 750 (Figures 6B1 and 6B2) and 1000 stimulus (Figures 6C1 and 6C2). For highly synchronized stimuli (σ_stim_/τ_*m*_ < 0.5), sharpening influenced by both variations noise correlation and the number of synapses comprising the total stimulus. This result is expected because with a greater number of spikes, the reliability of responses increases and resulting in a decrease in output dispersion.

## Discussion

In order to explore the interaction of short-term depression with neuronal firing dynamics in setting the operating mode of the neuron, we studied four canonical combinations of pre- and postsynaptic dynamics. Type 1 synapses show release-dependent depression and constant rate of recovery. They are capable of encoding the stimulus rate change in the response amplitude. Type 2 synapses, on the other hand show release-independent depression, and recover faster at higher rates. They are capable of maintaining substantial response amplitudes even at high stimulus rates. For the postsynaptic dynamics, we considered class 1 neurons that fire regularly and class 2 neurons, which exhibit spike-frequency adaptation. The first action potential response of all four possible combinations (T1C1, T1C2, T2C1 and T2C2) to a stimulus that was Gaussian distributed in time was characterized. We also investigated the sensitivity of these responses to correlations in background noise and to the number of synapses comprising the stimulus.

We found that the combination T1C2 can be characterized as a coincidence detector while the other three combinations were integrators each with specific features: T2C1 was an integrator with greatest reliability, T1C1 an integrator with greatest preservation of synchrony and T2C2 and integrator with greatest response sharpening. Specifically, the degree of reliability and preservation of synchrony varied across these integrators. The sensitivity to noise correlation and the extent of synaptic depression were different.

Though the results are based on simulations using models of dynamical synapses as well as neurons, we believe that our results capture the interactions realistically for the following reasons, Firstly, the synaptic dynamics are based on fitting the chosen model to EPSCs recorded in pairs of neurons *in vitro* (Scott et al., 2012). Individual EPSC peak conductances were set at 1 nS, a value that has been determined experimentally and modeled as alpha synapses with a decay time constant of 1 ms, which is similar to experimentally measured values (Stricker et al., 1996). In addition, varying the extents of type of classes did not systematically change the results in a qualitative sense (data not shown). We tested if it was indeed the adaptation current in class 2 neurons that produced the dynamics or whether an increased conductance of class 1 neurons might be sufficient to reproduce the effect. Increasing the conductance of a class 1 neuron did not reproduce operating modes that were obtained with class 2 neurons but produced responses that were qualitatively similar to those with class 1 neurons (data not shown). This is consistent with existing work that suggests that increase an in conductance converts class 2 into class 1 (Stiefel et al., 2008, 2009). Consequently, we think that our results robustly reflect the dynamics between type and class.

Secondly, the postsynaptic neuron had an effective neuronal time constant of 60 ms (in the presence of synaptic background noise), which is similar to experimentally measured values both *in vitro and vivo* (Destexhe et al., 2003). For the cell to fire a first action potential, typically about 45 synaptic events required to be activated within 10 ms. For class 2 neurons, the adapting current resembled a slow potassium conductance. There are two ways to interpret times of individual events that comprise the stimulus. The first is to consider them presynaptic spike arrival times. The second is to consider them presynaptic spike times. Propagation delays are not considered and hence, if the second interpretation is followed, precessions reported might be systematically overestimated. Timing of only the first spike was considered. Thus, our results are applicable in a context when the membrane potential of a class 1 or class 2 neuron is near threshold and presynaptic spikes are delivered through type 1 or type 2 synapses. In this study, information encoded in repetitive spiking is not considered as it is affected not only by incoming signal but also back-propagating action potentials and steady state dynamics.

### Emergent properties through interaction of pre- and post-synaptic dynamics

An important question to answer is if the properties observed were largely the result of either pre- or post-synaptic dynamics alone or if these combinations gave rise to emerging characteristics. We think the latter is the case for the following reasons. Considering presynaptic dynamics separately, the prediction might be that T1C1 and T1C2 are coincidence detectors while T2C1 and T2C2 are integrators. In addition, combinations with type 1 synapses will have reliable responses only when inputs are sufficiently synchronized and combinations with type 2 synapses will have reliable responses over a much higher range of stimulus dispersion. In contrast, considering firing dynamics separately, the prediction might be that T1C1 and T2C1 are integrators and T1C2 and T2C2 are coincidence detectors. Furthermore, combinations with class 1 neurons exhibit reliable responses over a wide range of stimulus dispersion and those with class 2 neurons require synchronous inputs. Since class 2 neurons have a slow hyperpolarizing conductance, stimuli have to be sufficiently short and strong to evoke a response before the slow conductance is activated and decreases the probability of an action potential. However, only some of these predictions are correct. For instance, T1C2 is a coincidence detector, but T1C1 is an integrator with greatest synchrony preservation, even though presynaptic dynamics remain the same. All four configurations have unique properties and hence not considering the contribution of either result in an incomplete view of neuronal encoding. Intuitively, T2C1 is expected to be the most effective integrator and it is indeed from the standpoint of reliability. But T1C1 is a more effective integrator from the standpoint of the relation between stimulus dispersion and response jitter. Stimulus dispersion is more effectively captured by the response dispersion. This can be viewed as a tradeoff between synchrony preservation and reliability.

Both pre- and post-synaptic dynamics contribute for a specific operating mode to emerge. Our results suggest that a complete characterization of neuronal encoding can be obtained only by considering both pre- and post-synaptic dynamics together.

There is evidence for matching of synapse type with firing class in the literature. For example, synapses in layer IV show target-specificity with spiny stellates receiving predominantly type 1 synapses and star pyramids and pyramids receiving predominantly type 2 synapses (Cowan and Stricker, 2004). Such specificity has also been reported in the lobster pyloric network where a disruption of specificity results compromised function (Mamiya and Nadim, 2005). Since each combination performs specific stimulus to response transformations, a slight change in either synapse type or neuron class can cause significant changes in information processing of individual neurons and within the network.

### Implications for synchronization and coding

The background noise correlation was found to be a critical determinant of response sharpening (ξ) as preservation of stimulus synchrony or its enhancement would have important consequences for processing at the network level. When ξ > 1, stimulus synchrony is enhanced by postsynaptic neurons and, thus, the firing becomes more synchronized as excitation is transmitted through subsequent layers (Marsálek et al., 1997). The signal becomes temporally sharpened while losing information about the stimulus dispersion (Gerstein et al., 1989). From the perspective of single neuron oscillations, if ξ is taken to indicate the relation between successive cycles of oscillation, discharges of neurons might become more synchronized (ξ > 1), conserve synchrony (ξ = 1) or progressively lose synchrony (ξ < 1). While previous studies have considered either synaptic dynamics (Mamiya and Nadim, 2005; McDonnell et al., 2012) or neuronal dynamics (Ermentrout, 1996, 1998; Marella and Ermentrout, 2008) in shaping oscillatory dynamics in networks, there was virtually no study exploring how these properties might together determine synchronization of individual neurons and consequently the network. In fact, we show that the combination T1C1 is best suited for preserving input synchrony. In this context, T1C1 might aid in the preservation of asynchrony in a network and might aid in encoding of network information through desynchronization (Hanslmayr et al., 2012). But, in general, network effects of integrator configurations are much harder to speculate about without performing detailed simulations since the larger time window of summation (when compared with the integrators) allows for possible interactions with feedback connections of a recurrent network and the timing of the second action potential might be modulated by network effects. Even so, our results for integrators do have relevance for network processing since sharpening (see T1C1: Preserves Synchrony Most Effectively and T2C2: Maximum Response Sharpening) and delay to fire first action potential (data not shown) will influence the overall network encoding.

### Type and class might enhance information processing

For the purpose of this paper, both synapse type and firing class were taken to be discrete properties. However, experimental evidence shows that type 1 and type 2 synapses exist along a continuum between release-dependence and release-independence and various experimental conditions can alter the extent of the release-dependence (Cowan and Stricker, 2004; Fuhrmann et al., 2004). Likewise, postsynaptic firing can vary smoothly between class 1 and class 2 properties (Stiefel et al., 2008, 2009). In addition, both the synapse type (unpublished data) and the firing class (Stiefel et al., 2008) can be altered concomitantly by neuromodulators like noradrenaline, and, thus, can be converted into each other. Further, there is intrinsic variability in firing dynamics among neurons of the same type (Schulz et al., 2006) that might be critical for maximizing information content (Padmanabhan and Urban, 2010). Our results suggest that variability in synapse type and firing class allows for specific neurons in the same network to capture and thereby encode different aspects of the stimulus. For instance, combinations with T1C2 properties would act as coincidence detectors. Upon exposure to a neuromodulator like noradrenaline, both type and class are converted to become more T2C1-“like” and as a consequence, the same node in the network would act now as an integrator with greatest reliability. Any partial conversion along type and/or class would allow for other features about the stimulus to be encoded. For instance, the combination of T1C2 (coincidence detector) might be converted to a reliable integrator (T2C1) by concomitant conversion of type and class due to adrenergic modulation. For the same condition, T1C1 (integrator with greatest synchrony preservation) would be converted to T2C1, an integrator with improved reliability but loss of synchrony preservation. Thus, neurons in a network might be tuned to capture and encode various stimulus properties of interest.

### Conflict of interest statement

The authors declare that the research was conducted in the absence of any commercial or financial relationships that could be construed as a potential conflict of interest.
